# A customizable and low-cost 3D-printed transwell device coupled with 3D cell culture for permeability assay

**DOI:** 10.1016/j.ohx.2024.e00603

**Published:** 2024-11-06

**Authors:** Pitaksit Supjaroen, Wisanu Niamsi, Pannawich Thirabowonkitphithan, Parichut Thummarati, Wanida Laiwattanapaisal

**Affiliations:** a Graduate Program in Clinical Biochemistry and Molecular Medicine, Department of Clinical Chemistry, Faculty of Allied Health Sciences, Chulalongkorn University, Bangkok 10330, Thailand; b Department of Biomedical Science, Faculty of Health and Society, Malmö University, 205 06 Malmö, Sweden; c Biofilms - Research Center for Biointerfaces, Malmö University, 205 06 Malmö, Sweden; d Centre of Excellence for Biosensors and Bioengineering (CEBB), Chulalongkorn University, Bangkok 10330, Thailand; e Department of Clinical Chemistry, Faculty of Allied Health Sciences, Chulalongkorn University, Patumwan, Bangkok 10330, Thailand

**Keywords:** Intestinal permeability, Invasion, Transwell-based assay, Paper membrane, 3D-printed transwell device, Permeability assay

## Abstract

The permeability-based assay is commonly used to assess intestinal barrier function, and it relies on using a transwell insert as an essential compartment. The device consists of a semipermeable membrane that is attached at the bottom of the insert and splits the system into the apical and basolateral compartments. However, commercial inserts are standardized with different pore sizes based on the application and offer only a flat plane of two-dimensional cell culture. Herein, we present a simple, low-cost 3D-printed transwell device and a robust method to functionalize the inserts for paper-based 3D cell culture. This 3D-printed device was fabricated from a polylactic acid (PLA) filament, and a paper membrane used to support HT-29 cells for intestinal permeability assessment. A device showed good biocompatibility when culturing HT-29 cells for 48 and 72 h with 97 % and 98 % cell viability, respectively. Together with fluorescence images, cells were attached directly to the microfiber networks of a Matrigel-functionalized paper, indicating that the functionalized paper is biocompatible and bioactive. Furthermore, in a more appropriate culture microenvironment, SEM analyses revealed cellular features differentiating into mucus-secreting cells, evidenced by the formation of microvilli on the cell surface, which was further confirmed by immunofluorescence staining of villin-1. To demonstrate the usability of the 3D-printed transwell device, intestinal permeability was assessed using both chemical and biological stimulation treatments. The permeability results employing FITC-dextran validated the association between a different level of relative fluorescence intensity unit (RFU) and the orange color of live cells by CellTracker^TM^. As a result, this 3D-printed transwell device provides a straightforward and cost-effective method for manufacturing a device for customization in many laboratory settings, making it a feasible alternative to marketed transwell devices that do not allow for customization.

## Graphical abstract

**Figure:**
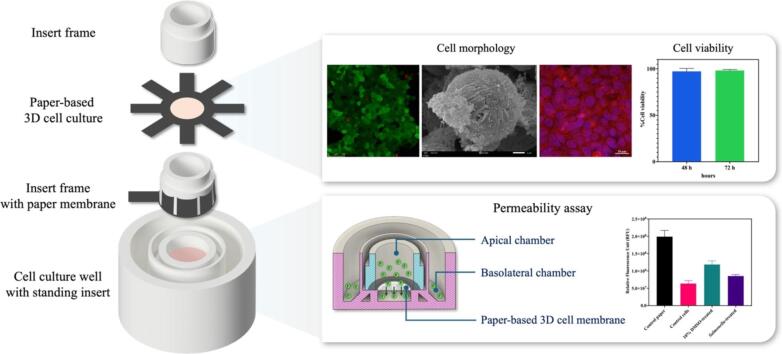

**Specifications table**

**Table:** 

Hardware name	3D-printed transwell device
Subject area	• Engineering and materials science • Chemistry and biochemistry • Medical; biomedical engineering, pharmaceutical science, infectious disease • Biological sciences; microbiology and biochemistry
Hardware type	• Biological sample handling and preparation
Closest commercial analog	Transwell cell migration and invasion assay
Open source license	CC BY 4.0
Cost of hardware	∼0.18 USD
Source file repository	https://doi.org/10.17632/vgdth6ytk5.1

## 1 Hardware in context

The transwell-based assay is a standard method and the most widely used cell culture platform to investigate cell behaviors and other biomedical applications such as cell migration, cell invasion, cell permeability, cancer biology, and drug discovery [1], [2]. Intestinal permeability is a physiological structure of the human intestine system. The transwell-based assay can help researchers study and understand the transportation of substances across the paracellular route of intestinal permeability and examine biological processes of cellular mechanisms, cell-to-cell interactions, host-microbiome interactions by single-cell culture, and co-culture of various cell types. The most common cell lines of intestinal epithelial cells (IECs), for example, Caco-2 and HT-29 cells were used for *in vitro* study with their characteristics and functions [3]. In addition, to study the host-microbiome interaction, *Salmonella* is one of the invasive pathogens with the ability to adhere and invade the host cell. The effect of *Salmonella*-infected cells is to disrupt intestinal permeability and lead to cell damage [3].

One crucial component of the transwell insert is the semipermeable membrane, which splits the system into an apical and basolateral compartment. A semipermeable membrane of the transwell insert is typically fixed with a flat plane of polycarbonate (PC), polyethylene terephthalate (PET), and polyester (PE) membranes with defined pore sizes depending on the applications [3], [4], [5]. These membranes promote cell growth in two-dimensional (2D) shapes and have no anatomical relevance to the native morphology and environment. Therefore, 3D cell culture models have been developed by exploring biocompatible materials that are suitable to fabricate a scaffold membrane for the 3D microenvironment. Currently, a commercially available 3D scaffold membrane insert made from polystyrene is on the market. It has been demonstrated for use in co-culture models of intestinal epithelial cells, as well as for supporting other cell types. [6]. Additionally, electrospinning is an alternative method that can be used to fabricate scaffold membranes for 3D cultures of intestinal epithelial cells [7]. The method can define the pore size of scaffold fibers in micro- and nanoscale. However, this method requires a special instrument and laboratory skills, and cytotoxicity might be affected by the residual solvent from the electrospinning process [8]. These membranes are supported by a plastic insert frame, which may limit their use for specific purposes. Alternatively, paper membranes offer a low-cost, easy-to-fabricate, and biocompatible commonly used in laboratories. They have been widely applied in biological applications, such as paper-based transwells [9] and paper chips of intestinal cell culture for permeability assessment. According to the morphology properties of paper membranes, the microfiber network is a cellulose that can be modified with a Matrigel matrix to mimic the native extracellular matrix (ECM) [10]. A Matrigel-functionalized paper is suitable for constructing the 3D microenvironment of a basement membrane in the intestinal permeability.

A 3D printing technology is a well-known and popular technique for the production of robust, safe, and low-cost biological platforms in various applications as well as the transwell-based assay, for example, 3D-printed integrated scaffold-based cell culture for transepithelial electrical resistance (TEER) measurements [11], 3D-printed integrated electrochemical sensors [12], 3D-printed cell culture insert [13]. Interestingly, Polylactic acid (PLA) offers an eco-friendly and biocompatible polymer that is widely used as a filament for 3D-printed models with multiple purposes in biomedical applications [14], [15], [16].

Here, we present a customizable and low-cost 3D-printed cell culture chamber and transwell insert coupled with 3D cell culture using a Matrigel-functionalized membrane. This study aims to design and develop a unique, open-source, and robust method of manufacturing 3D-printed transwell devices of the transwell-based assay for intestinal permeability assessment employing a 3D intestinal cell model and FITC-dextran assay [17], [18]. This device offers a simple, sustainable, and cost-effective solution for cell and tissue engineering in disease modeling, drug screening, and drug discovery.

## 2 Hardware description

Commercial transwell inserts do not allow custom design and size. In this work, 3D printing with PLA filament offers a customizable method to create the cell culture chamber and insert frame for the permeability assay with simple, low-cost, and suitable alternative membranes. The device consists of three main components: 1) a well with a standing insert, 2) an insert frame, and 3) a wax-patterned functionalized paper. The dimension of the device was closely matched to a standard 24-well cell culture plate.

Advantages:

• A 3D-printed transwell device is a customizable device that can be future adjusted to other standard sizes such as 6-well and 12-well which is suitable for the proposed applications.
• 3D printing technology is a cost-effective method to manufacture the transwell-based device when compared to commercially available transwell inserts.
• A 3D-printed transwell device coupled with a paper membrane insert that is made up of a biocompatible filament and a network of cellulose that is a greener material when compared to plasticware with a good responsibility for sustainability.
• Utilize a single-separated well to avoid wasting and retain the sterilization of other wells on the entire plate that are not in use.
• Minimize the potential risks when removing and harvesting the membrane for future investigation using forceps and it is possible to stack the paper for co-culture with other cell types.

***Design files***

**Adjustable 3D printed files**

These 3D-printed cell culture cup models were created using Autodesk Fusion 360 software. The key benefit of this software lies in its changeability, allowing users to easily adjust the size parameters of both the cell culture well cup housing and insert. Through modification of parameters in the Autodesk Fusion 360 Archive File (.f3d) format, users can adjust and modify in terms of diameter, width, and height to be compatible with the cell culture chamber and insert frames in different sizes. These f3d files are editable within the Autodesk Fusion 360 software. Detailed instructions for modifying these parameters can be found in section 5 (build instructions).

1. Well with standing insert.stl

**Fig. 1:**
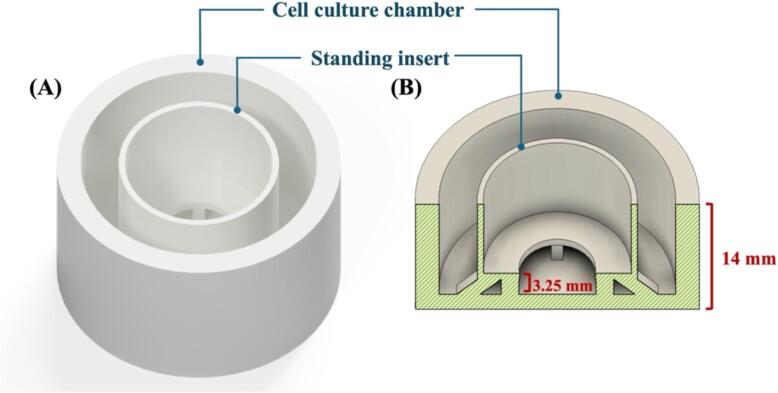
Schematic illustration of (A) Overview and (B) cross-section view of a 3D-printed transwell device.

The file includes a 3D-printable model of a customizable cup and standing insert that can be adjusted using Autodesk Fusion 360. In this study, we utilized the design of 24 mm in diameter and 14 mm in height, with a 3.25 mm gap size between the bottom of the cell culture well and the standing insert where the membrane is placed as shown in Fig. 1.

2. Insert Frame.stl

**Fig. 2:**
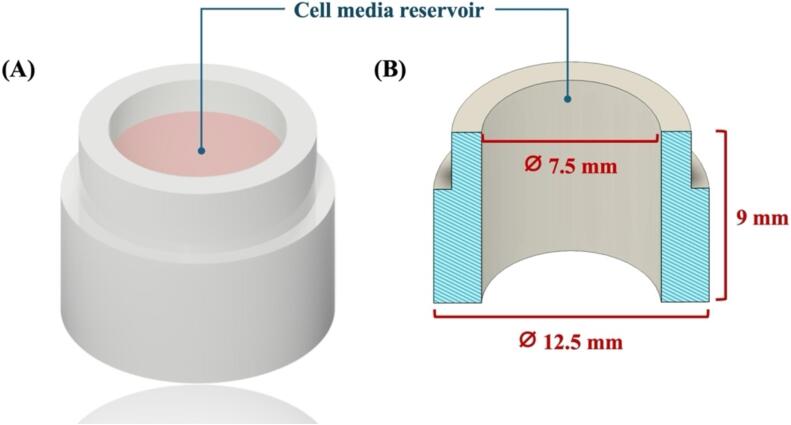
Schematic illustration and dimension of a 3D-printed insert frame.

The dimensions of an insert frame are 7.5 mm in diameter and 9 mm in height as shown in Fig. 2. This component is closely matched with a transwell insert for a standard 24-well cell culture plate. The working volume of the cell reservoir is up to 0.3 ml.

3. Wax-patterned paper.ppt

**Fig. 3:**
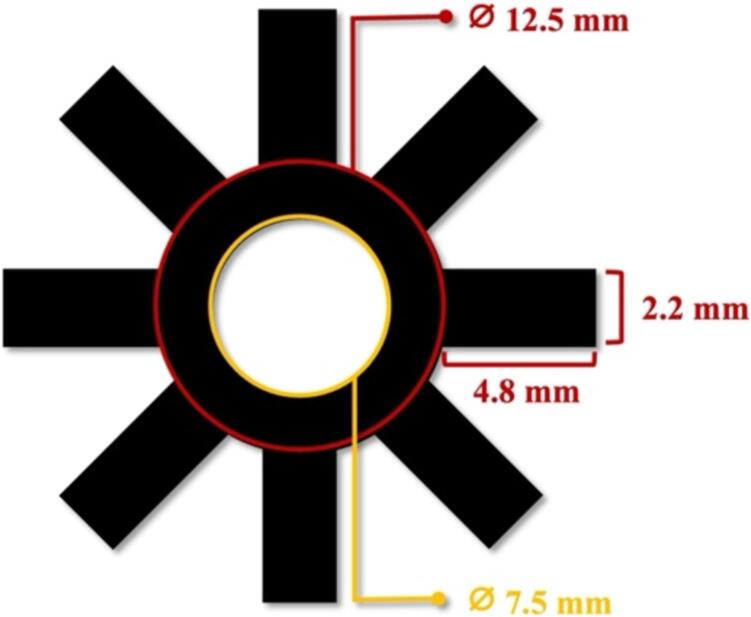
Schematic drawing and dimension of a wax-patterned paper.

The design of the wax-patterned is a star or asterisk (*) symbol with a circular shape inside, with a diameter of 7.5 mm (surface of culture area = 0.44 cm^2^) as a hydrophilic area that will be used for cell culture as shown in Fig. 3. The dimensions of the wax pattern were designed to fit with the insert frame and cell culture chamber.

## 3 Design files summary

**Table:** 

Design file name	File type	Open source license	Location of the file
Original dimension			
Well with standing insert	STL	CC-BY 4.0	https://doi.org/10.17632/vgdth6ytk5.1
Insert frame	STL	CC-BY 4.0	https://doi.org/10.17632/vgdth6ytk5.1
Wax-patterned paper	PPT	CC-BY 4.0	https://doi.org/10.17632/vgdth6ytk5.1

## 4 Bill of materials summary

**Table:** 

Designator	Component	Number	Cost per unit (USD)	Total cost (USD)	Source of materials	Material type
**Components of a 3D-printed transwell device**						
3D-printed transwell devices (Well with standing insert)	3D printer filaments (PolyLite PLA Pro, white filament spool, 1.75 mm diameter)	1	0.08	0.08	Amazon	Polymer (PLA)
Insert frame	3D printer filaments (PolyLite PLA Pro, white filament spool, 1.75 mm diameter)	1	0.01	0.01	Amazon	Polymer (PLA)
Semi-transparent film	Parafilm	1	0.01	0.01	Amazon	Flexible covering sealing film
Membrane	Whatman Grade 1 filter paper	1	0.003	0.003	Cytiva	Cellulose fibers
Wax-patterned	Wax ink	1	0.005	0.005	Xerox	Solid ink
Extracellular Matrix (ECM)	Matrigel matrix	1	0.075	0.075	Corning	Solubilized basement membrane

The dimensions of a 3D-printed transwell device closely match to 24-well standard size. The cost of parafilm is less than 0.01 USD per device [19], a wax-printed on paper costs 0.001 USD per device of 1 cm^2^, the solid ink is approximately 0.0001 USD per cm^2^ and a standard filter paper is approximately 7.00 USD per m^2^ [20]. The costs of all components of 3D-printed devices were calculated into USD units for a single piece and a pack of 48 pieces, which is 0.18 USD and 8.64 USD, respectively. This data offers an interesting method for manufacturing a transwell-based device for alternative membranes using 3D printing with a low-cost solution.

## 5 Build instructions

### 5.1 Fabrication of 3D-printed cell culture device

**Fig. 4:**
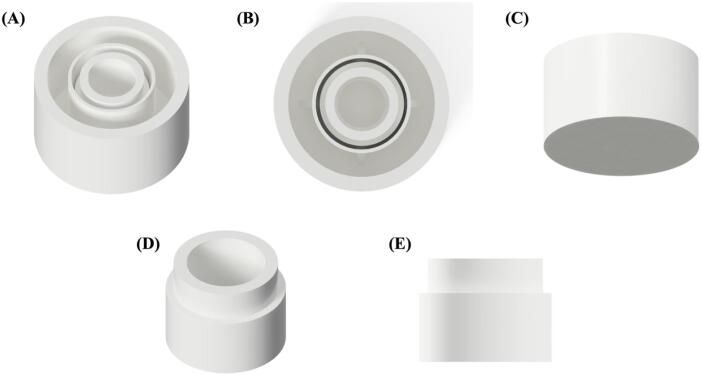
Schematic illustration of a 3D-printed transwell device; (A-C) Top and bottom views of a well with standing insert, (D, E) Top and side views of an insert frame.

All three parts of the cell culture device were printed using a fused deposition modeling (FDM®) or fused filament fabrication (FFF) 3D printer, which is cheaper and more customizable than a resin 3D printer. Various types of filaments can be chosen based on different applications. For certain plastic materials such as Acrylonitrile Butadiene Styrene (ABS), Acrylonitrile Styrene Acrylate (ASA), Polyethylene Terephthalate Glycol (PETG), and Polypropylene (PP), filament often works well for high temperatures and offers more strength than Polylactic Acid (PLA) filament. However, PLA, PETG, and PP are more biocompatible than ABS and ASA, making them suitable for cell culture purposes. In this study, we chose PLA filament for the device due to its biocompatible properties, cost-effectiveness, and ease of printing compared to other filaments [14]. The devices can be easily optimized for each part, such as the gap between the bottom of the well and the membrane in the standing insert or optimizing the cell culture area (diameter). The main body of the cell culture cup is designed to be printed in place a printed without the need for any further manual assembly, combining the well plate and standing insert in one print to maintain a simple setup and optimize the printing process (Fig. 4).

***Printing steps***

SAFETY HAZARDS

The following safety instructions must be followed during the 3D printing.

- Always use personal protective equipment (PPE) during operations.
- Make sure to check the filament's compatibility before running any experiments. Due to different brands and types of filament, some filaments may produce toxic fumes or hazardous particles such as volatile organic compounds (VOCs) and particulate matter (PM) [21]. Printing in an open-air environment is recommended.
- If the printer lacks safety detection features, always keep monitoring the printing process; do not leave the printer running without someone observing.

In this model, polylactic Acid (PLA) filament (PolyLite PLA Pro, Shanghai, China) was utilized to fabricate the cell culture device using a locally custom-built FFF 3D printer (i3Dbot x250 3D printer, Thailand). All 3D printing files were sliced using the open-source PrusaSlicer 2.7.1 (Prusa Research, Prague, Czech Republic) and then exported as geometric code (.gcode) files to the 3D printer. All parameters recommended for printing with PLA filament are provided in Table 1. The well with standing insert and insert frame was printed without using support (files Well with standing insert.stl and insertframe.stl, respectively).

**Table 1:** Recommended printing parameters.

Printing Parameter	Value/Unit
Material	1.75 mm PLA filament
Printing Temperature	195–220 °C
Bed Temperature	60 °C
Layer Height	0.2 mm
Perimeter/vertical shells	2
Infill Percentage	15 %
Perimeter Speed	70 mm/s (depend on 3D-printer hardware)

### 5.2 Wax-patterned paper

**Fig. 5:**
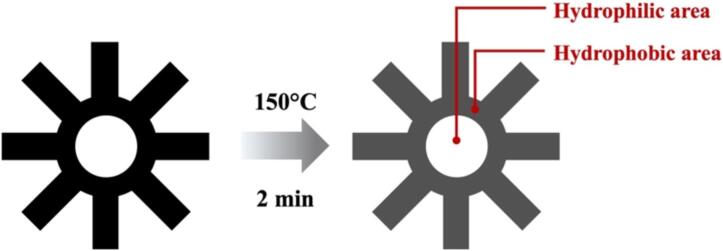
Schematic representation of a wax-patterned paper.

The Whatman grade 1 filter paper (Cytiva, USA) was used as a scaffold for the cell culture platform. The wax pattern was designed by PowerPoint software (PowerPoint, Microsoft Corporation, Redmond, WA, USA) and printed with a wax printer (ColorQube 8570, Xerox Corporation, Norwalk, CT, USA). After that, the wax-patterned paper was heated at 150 °C for 2 min on a hot plate to allow wax ink to fully penetrate through the thickness of the porous membrane, creating a hydrophobic pattern and leaving a circular hydrophilic area on the paper, as shown in Fig. 5. The paper was then allowed to cool down before being cut into the desired shape. For safety, be careful when working with the hot surface of the hot plate while heating wax-printed paper. Finally, a wax-patterned paper was exposed to ultraviolet (UV) radiation for 15 min on each side before being used for the cell culture application.

Although the wax printer (Xerox ColorQube) is no longer available on the market, the wax-patterned paper can be fabricated using alternatives commercial wax ribbon or a custom-made ink ribbon with a thermal transfer printer [22], [23]. These methods can facilitate the direct transfer of patterned wax onto the paper, creating hydrophobic and hydrophilic barrier, and potentially used as paper substrate for 3D cell culture applications.

## 6 Operation instructions

### 6.1 The co-culture model of intestinal HT-29 cell line and Salmonella Typhimurium bacteria

The human colorectal epithelial cell line HT-29 was used as a cell model of intestinal permeability. Cells were cultured in Dulbecco’s Modified Eagle (DMEM) medium supplemented with 10 % fetal bovine serum (FBS) (Sigma, USA) and 1 % Penicillin/streptomycin at 37 °C and 5 % CO_2_ incubator. When cells reached 80–90 % confluency, the cell suspension was prepared by trypsinization using 0.25 % trypsin-EDTA solution. Then cells were centrifuged at 1,500 rpm at 25 °C for 5 min.

The inoculated suspension of *Salmonella* Typhimurium was grown in Lysogeny broth (LB) medium in an orbital shaker incubator at 37 °C. For cell infection, bacteria were centrifuged at 3,260 g at 4 °C for 10 min. Then the quantification of microbes was diluted in a culture medium and prepared to the multiplicity of infection (MOI) at 100 by measuring the optical density at 600 nm.

### 6.2 Assembly of a 3D-printed transwell device incorporating a paper membrane

1. All components of 3D-printed devices were sterilized with 70 % ethanol solution for 15 min and dried inside the biosafety cabinet (BSC) class II.
2. The devices were exposed to ultraviolet (UV) radiation for 15 min each side.
3. After completing the sterilization, a wax-patterned paper was attached to the insert frame and then sealed with parafilm as shown in Fig. 6A.

**Fig. 6:**
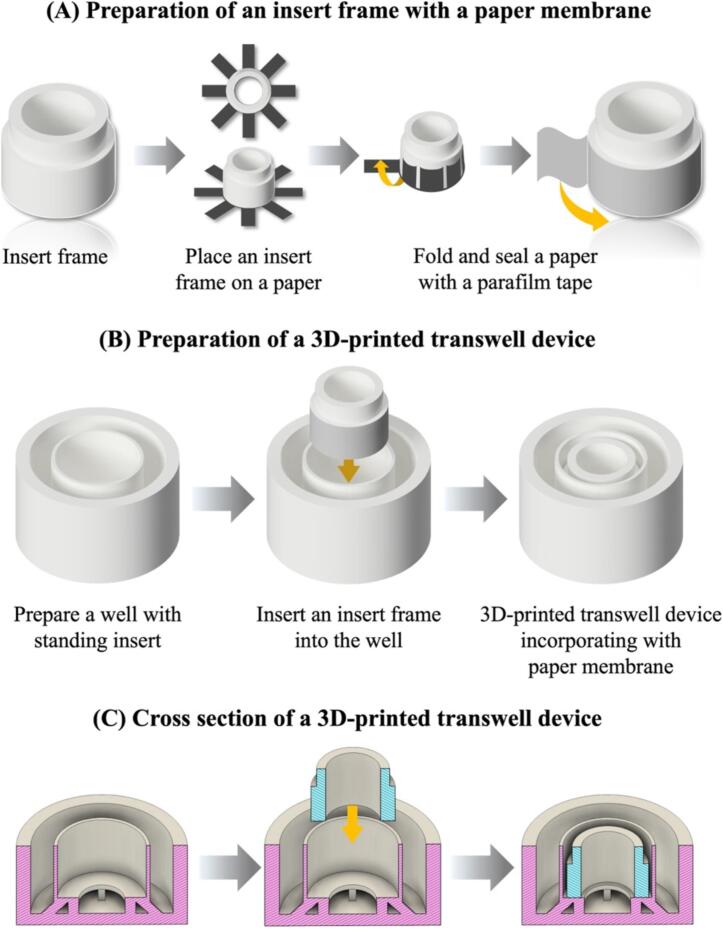
Operation instructions of a 3D-printed transwell device; (A) Fabrication of a wax-patterned paper with an insert frame, (B) Insertion of an insert frame into the well, and (C) cross-section view of a 3D-printed transwell device.
4. Join together the insert frame with a wax-patterned paper into a 3D-printed transwell device as shown in Fig. 6 (B, C).
5. Coating a wax-patterned paper with 10 µL of Matrigel matrix solution and then gelatinized at 37 °C for 30 min.
6. Rinse twice with pre-warmed PBS to eliminate any remains.
7. Then, add 1.50 mL of culture medium to the basolateral compartment, followed by 0.19 mL in the apical compartment.
8. Seed 0.19 mL of the cell suspension at 8.0 × 10^5^ cells/well (4.2 × 10^6^ cells/ml).
9. Allow the seeded cells in 3D-printed transwell devices to settle for 5 min before transferring to a 37 °C, 5 % CO_2_ incubator.

### 6.3 Investigation of cell viability, barrier formation, and morphology on the 3D-printed device

*Fluorescence imaging*

The phenotypic characteristics and viability of cells cultivated on a 2D culture plate, and a 3D paper membrane were compared using the LIVE/DEAD dyes. The HT-29 cells were seeded at a density of 8.0 × 10^5^ cells per well for both conditions. Following 48 h of culture, cells were washed and stained with 4 µg/ml of calcein-AM solution and 10 µg/ml of propidium iodide (PI) at 37 °C for 20 min [24]. The stained cells were washed with 1X PBS and placed on a glass plate for fluorescence imaging with a confocal scanning microscope (ZEISS LSM 980, Germany). Following characterization, the vitality of HT-29 cells on a 3D-printed transwell device was assessed using the same method. After 48 and 72 h of growth, the paper-based HT-29 cell membranes were washed with PBS and incubated with 4 µg/ml of calcein-AM solution and 10 µg/ml of propidium iodide (PI) for 20 min at 37 °C [24]. The calcein-AM/PI-stained cells were gently rinsed with PBS before being placed on a glass plate for fluorescence imaging. Cell imaging was performed with a confocal laser scanning microscope (Leica DMi8, Germany) and a 40X objective lens. The fluorescence intensity was determined from five fields of captured images using Image J software [25]. The average intensity of green (live cells) and red (dead cells) channels were calculated to the percentage of cell viability as follows:

$$Cell\;viability\left(\%\right)\;=\;\frac{Live\;cells}{\left(Live\;cells+Dead\;cells\right)}\times 100$$

**Fig. 7:**
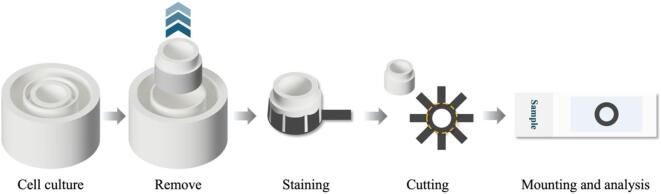
Preparation of a wax-patterned paper for the biological investigation.

To investigate cell barrier characteristics of HT-29 cells on a Matrigel-functionalized membrane using CellTracker^TM^ Orange dye (Invitrogen, USA). HT-29 cells were stained by incubated with CellTracker^TM^ Orange in a serum-free medium for 20 min at a 37 °C incubator [26]. After that, cells were washed with a culture medium and followed by resuspended with the culture medium. Cells were seeded on a Matrigel-functionalized membrane of a 3D-printed transwell device and cultured for 48 h, then treated with different conditions as described in the conditions of the permeability assay. For cell imaging, paper membranes were transferred to glass slides as shown in Fig. 7, and images were captured with BioTek Cytation 7 cell imaging multimode reader (Agilent, USA). Cell images were observed using wavelengths of excitation and emission at 548 and 576 nm, respectively [26].

*Scanning electron microscopy (SEM)*

To investigate the physical morphology of HT-29 cells on a paper-based membrane after 48 h of culture, the sample was fixed and preserved with 2.5 % glutaraldehyde in cold PBS. The sample was then sputter-coated with gold and then examined with a field-emission scanning electron microscopy (FESEM) (JEOL, model JSM7610F, Japan).

Furthermore, immunofluorescence staining was performed to investigate the surface villi of HT-29 cells on a Matrigel-functionalized membrane using immunofluorescence staining. Cells were fixed with 4 % paraformaldehyde (PFA) phosphate buffer solution for 20 min at room temperature (RT). After that, the fixed cells were washed 3 times with PBS and permeabilized with 0.5 % Triton X-100 at RT for 30 min. Then the cells were washed 3 times with PBS and blocked with bovine serum albumin (2 % BSA in PBS) at RT for 90 min. Next, the cells were incubated with the primary antibody of Mouse monoclonal Anti-Villin-1 (1:200) at 4 °C overnight. After that, the cells were washed 3 times with PBS and then incubated with secondary antibodies of Alexa Fluor 594-conjugate Anti-mouse IgG (1:2000) antibodies for 1 h. Lastly, the cells were washed 3 times with PBS for 5 min and incubated with Hoechst 33342 (10 µM) solution at RT for 1 h. The stained cells were observed under a confocal laser scanning microscope (ZEISS LSM 980, Germany).

*Permeability assay*

A permeability assay of the intestinal cells was performed using Fluorescein isothiocyanate-carboxymethyl-Dextran (FITC-Dextran, MW 10 kDa) [9]. After cultured HT-29 cells on a Matrigel-functionalized membrane for 48 h, the culture chamber was replaced with different conditions: (i) control paper contains only phenol red-free medium without cells, (ii) control cell contains phenol red-free medium with HT-29 cells, (iii) cells were treated with 10 % dimethyl sulfoxide (DMSO) in phenol red-free medium, and (iv) cells were treated with *S.* Typhimurium at 100 of multiplicity of infection (MOI) in phenol red-free medium. And all conditions also suspension with FITC-dextran dye.

After treatment, cells were incubated at 37 °C incubator for 4 h. To investigate the fluorescence intensity of FITC-dextran, Aliquots of 100 μL culture medium was collected from a basolateral compartment and transferred to a 96-well black flatted-bottom plate. The fluorescence intensity was measured at the wavelength of excitation 490 and emission 520 nm using SpectraMax iD3 Multi-Mode Microplate Reader (Molecular Devices, California). The solution of phenol red-free medium was used as a blank.

## 7 Validation and characterization

### 7.1 Biocompatibility

Both the 3D-printed cell culture chamber and insert frame were made of polylactic acid (PLA) filament and coupled with a Matrigel-functionalized membrane of Whatman grade 1 filter paper for 3D cell culture. To investigate the biocompatibility of the device, HT-29 cells were seeded at an initial density of 8.0 × 10^5^ cells/well on the Matrigel-functionalized membrane and HT-29 cells were cultured for 48 and 72 h. After that, viable cells were stained with calcein-AM, and dead cells were stained with PI staining dye and then investigated by the confocal microscope.

**Fig. 8:**
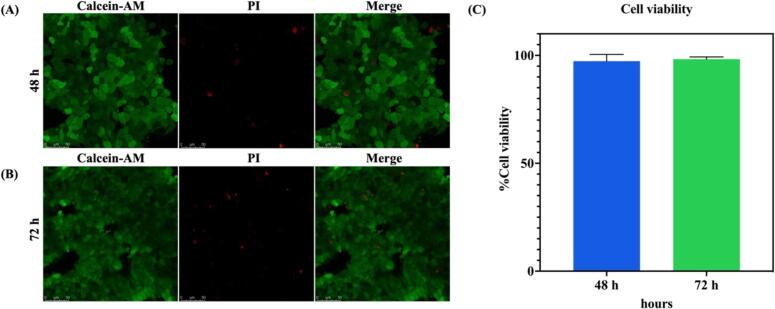
Immunofluorescence images of cell viability of HT-29 cells on paper membranes of 3D-printed transwell devices after culturing for (A) 48 and (B) 72 h. (Calcein-AM is green and Propidium iodide (PI) is red) The scale bar is 50 µm. And (C) The bar graph of the percentage of cell viability assessment. Data are presented as the mean ± standard deviation (SD) (n = 3). (For interpretation of the references to color in this figure legend, the reader is referred to the web version of this article.)

The fluorescence images showed that cells were attached to the cellulose fibers of a Whatman grade 1 filter membrane. As shown in Fig. 8 (A, B), the fluorescence signals of single-plane images were expressed at varying intensities, suggesting that cells were growing in distinct layers. The percentage of cell viability was calculated using the fluorescence intensity from fluorescence images. As shown in Fig. 8C, the cell viability after 48 and 72 h of culturing were 97 % and 98 %, respectively. Consequently, the system of a 3D-printed transwell device that incorporates a paper-based membrane modified with Matrigel matrix has minimal effect on the survivability of the cells and can be used as a 3D cell culture platform for the permeability assessment. Furthermore, we investigated the phenotypic morphology and viability of HT-29 cells cultivated on a 2D cell culture plate and a 3D paper membrane for 48 h. The results are shown in supplementary Figure S.1.

### 7.2 Cell morphology

A wax-patterned paper was modified with a Matrigel matrix that is a biochemical cue and mimics a native extracellular matrix (ECM) of the basement membrane to improve the 3D microenvironment and promote the differentiation of intestinal epithelial cells [27]. We investigated the physical morphology of a wax-patterned paper using scanning electron microscope (SEM) imaging.

**Fig. 9:**
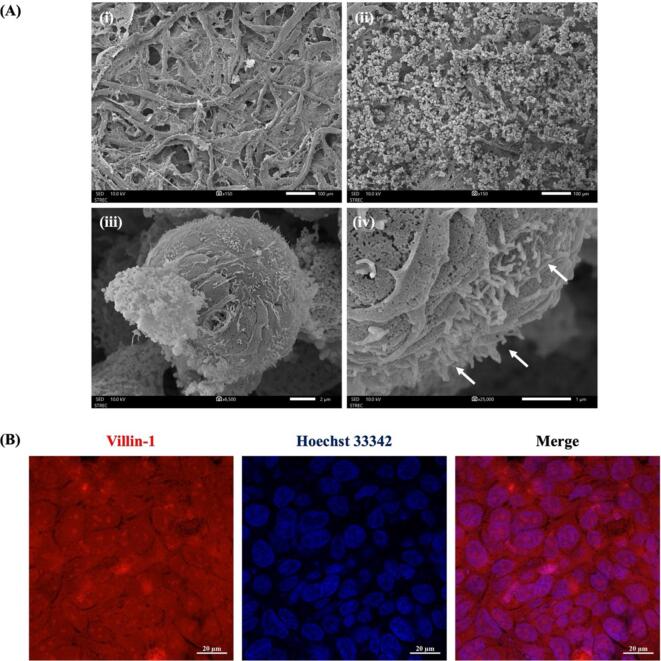
(A) SEM images of (i) Matrigel-modified Whatman grade 1 filter paper without HT-29 cells; (ii) with HT-29 cells, scale bar is 100 μm; (iii) HT-29 cells in a 3D shape, scale bar is 2 μm; and (iv) microarchitecture of microvilli structure (white arrow) on the cell surface in the normal condition, scale bar is 1 μm, and (B) Representative fluorescence images of villin-1 and Hoechst 33342 staining of HT-29 cells. The scale bar is 20 µm.

As shown in Fig. 9A(i), the SEM image of a Matrigel-modified paper without cell culture showed the surface of the entire microstructure of the fiber network coated with ECM proteins that a component of the Matrigel matrix such as laminin, collagen IV, and entactin [10].

Next, cells were seeded and cultured on a Matrigel-modified paper. As shown in Fig. 9A(ii-iii), cells were attached to the microfiber networks of a Matrigel-modified paper and formed in a 3D morphology. Furthermore, HT-29 cells are mucus-secreting cells with their characteristic high expression and release of mucin that can produce the mucus layer of intestinal permeability [28]. After culturing for 48 h, the SEM image showed that the microvilli appeared on the cell surface as shown in Fig. 9A(iv) (white arrow), indicating that cells started to differentiate and produced the mucus layer. Consistent with the representative fluorescence images, the red signal of villin-1 expression exhibited a continuously well-defined pattern on an apical surface as shown in Fig. 9B, indicating the microvilli of HT-29 cells.

### 7.3 Permeability assay

**Fig. 10:**
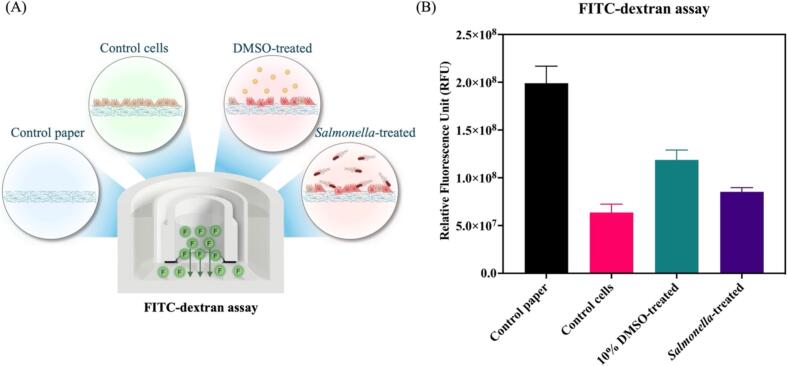
The permeability assessment of HT-29 cells on paper-based membranes of 3D-printed transwell devices after culturing for 48 h and treatment with different conditions. Data are presented as the mean ± standard deviation (SD) (n = 3). (Personal creation elaborated with Biorender.com).

To demonstrate the permeability assay of a 3D-printed transwell device coupled with 3D cell culture, we investigated the intestinal permeability of HT-29 cells at different conditions using the FITC-dextran assay. HT-29 cells were cultured on a Matrigel-functionalized membrane for 48 h and then investigated the permeability by incubating the conditioned medium containing FITC-dextran tracer for 4 h (Fig. 10A) [9]. After that, the culture medium was collected from the basolateral compartment and transferred to a 96-well plate for fluorescence intensity measurement.

A FITC-dextran assay is a tracer for the investigation of paracellular transportation [17]. The results showed that the relative fluorescence intensity (RFU) of a control paper without HT-20 cells was the highest as shown in Fig. 10B, indicating that FITC-dextran can pass through the microfiber network and porous of a wax-patterned paper modified with Matrigel matrix. The RFU of the cells without any treatments as control cells was decreased when compared to the control, suggesting that the presence of cells on the paper membrane reduced the transport of FITC-dextran from the apical compartment which confirm the formation of the barrier function.

In contrast, DMSO is a common chemical reagent that is used in several biological applications, especially, cell culture applications. At a high concentration and long exposure time, DMSO will affect the cell viability which increases cell damage. [29] In this study, cells were exposed to 10 % (v/v) of DMSO in a culture medium for 4 h. The results showed that the RFU of DMSO-treated cells was higher than control cells as shown in Fig. 10B, suggesting that DMSO-induced intestinal cell damage. Moreover, a high concentration of DMSO degrades ECM components and alters the mechanical properties of the Matrigel-functionalized membrane which causes the tracker of FITC-dextran to pass through the thickness of a paper membrane from the apical to the basolateral compartment.

Furthermore, we have demonstrated the host-enteric pathogen interaction using *S.* Typhimurium as an invasive pathogen. Previously, the investigation of invasion assay of *Salmonella*-infected HT-29 cells suggested that *S.* Typhimurium plays a role in tight junction disruption [3] that leads to loss of their interaction between cell to cell. In this study, cells were co-cultured with *S.* Typhimurium at MOI 100 for 4 h by adding the suspension of *S.* Typhimurium into the apical compartment (Fig. 10A). The RFU of *Salmonella*-treated cells was higher than control cells but lower than DMSO-treated cells as shown in Fig. 10B, indicating that *Salmonella*-infected HT-29 cells disrupted the cell–cell interaction that leads to increased FITC-dextran leakage from the apical compartment to the basolateral compartment. Additionally, the FITC-dextran assay of all conditions was measured at 1, 2, and 4 h post-incubation. The results are shown in supplementary Fig. S2.

### 7.4 Cell barrier characteristics

**Fig. 11:**
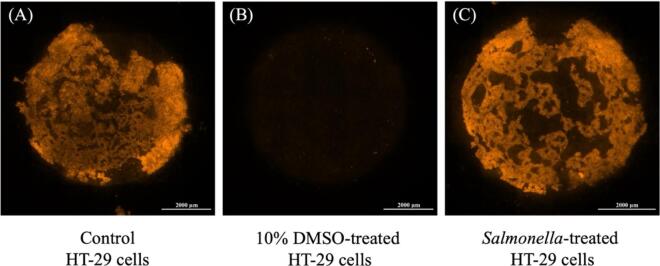
Fluorescence images of HT-29 cells on a paper-based membrane with different conditions; (A) HT-29 cells without any treatment, (B) HT-29 cells with 10 % DMSO treatment for 4 h, and (C) HT-29 cells with *S.* Typhimurium treatment at MOI of 100 for 4 h. The scale bar is 2,000 μm.

To investigate the cell barrier characteristics on a Matrigel-functionalized membrane of a 3D-printed transwell device, HT-29 cells were incubated with CellTracker^TM^ Orange dye and cultured for 48 h. Then HT-29 cells were treated with the same conditions as the permeability assay. The fluorescence signals of viable cells were expressed in the orange color as shown in Fig. 11 (A-C).

In the control group of HT-29 cells, cells were formed as an intestinal epithelial cell layer on a paper membrane as shown in Fig. 11A. Moreover, cells exhibited different fluorescence intensities on a paper membrane, indicating cells were infiltrated and attached to the microfiber network of a paper membrane in different layers.

In the group of 10 % DMSO treatment, cells exhibited a low fluorescence signal when compared to the control group, as shown in Fig. 11B. The result suggests a high concentration of DMSO treatment induced cell damage and altered the mechanical properties of a Matrigel-functionalized membrane. Therefore, no viable cells can be detected in this condition.

Furthermore, we investigated HT-29 cells on a Matrigel-functionalized membrane treated with *S.* Typhimurium at MOI 100. Consistent with the FITC-dextran assay, the fluorescence image showed that cells were formed looser than control cells, especially in the middle field of the entire surface as shown in Fig. 11C, indicating that infected cells were invaded and disrupted by *S.* Typhimurium. In addition, a high concentration of MOI promotes the adhesion ability of the bacteria. The adhesion of *S.* Typhimurium plays a vital role in cell survival, and cell replication [30], and they can penetrate the mucus layer of intestinal permeability by flagella-driven swimming [31] and directly bind to specific surface receptors of the host cells, followed by transport the bacterial protein into the cells. These results were consistent with the permeability assay as shown in Fig. 10B.

## 8 Conclusion

To fabricate a 3D-printed transwell device coupled with 3D cell culture for intestinal permeability assessment, in this work, we have presented a customized and low-cost 3D-printed cell culture chamber with an insert frame. The design and dimensions of this device are closely matched to a commercial transwell insert of a standard 24-well plate. The device is made from PLA filament which is eco-friendly and greener than available plasticware products. This device is available for a Matrigel-functionalized paper and is suitable for the 3D microenvironment of intestinal epithelial cell culture. Interestingly, the paper membrane was simple to remove and harvest from the device for biological investigations. Furthermore, we have comprehensively investigated cell morphology, cell viability, and cell barrier formation. Thus, a Matrigel-functionalized paper allows HT-29 cells to attach, proliferate, and differentiate into mucus-secreting cells. Here, we successfully demonstrated the permeability assay of HT-29 cells using a 3D-printed transwell device incorporating a paper membrane. After 4 h of FITC-dextran incubation, FITC-dextran can pass through the microfiber network of a Matrigel-functionalized membrane both with and without HT-29 cell conditions. Lastly, our device offers an alternative method that can be developed with more complexity to model the intestinal barrier function and intestinal permeability using different paper types as well as a co-culture model of intestinal epithelial cells and other cell types for high throughput of disease modeling, drug screening, and drug discovery.

## CRediT authorship contribution statement

**Pitaksit Supjaroen:** Writing – original draft, Methodology, Investigation, Conceptualization. **Wisanu Niamsi:** Writing – original draft, Methodology, Conceptualization. **Pannawich Thirabowonkitphithan:** Writing – review & editing, Methodology, Investigation. **Parichut Thummarati:** Visualization, Methodology. **Wanida Laiwattanapaisal:** Writing – review & editing, Supervision, Resources, Methodology, Funding acquisition, Conceptualization.

## Declaration of competing interest

The authors declare that they have no known competing financial interests or personal relationships that could have appeared to influence the work reported in this paper.
