# Supplementary material for: A customizable and low-cost 3D-printed transwell device coupled with 3D cell culture for permeability assay
Source: HardwareX. 2024 Nov 6;20:e00603. doi: 10.1016/j.ohx.2024.e00603 (PMC11585668; doi:10.1016/j.ohx.2024.e00603)
Supplement: Supplementary Data 1 [file mmc1.docx]

*Supplementary data*

**A customizable and low-cost 3D-printed transwell device coupled with 3D cell culture for permeability assay**

Pitaksit Supjaroen ^a^, Wisanu Niamsi ^a^, Pannawich Thirabowonkitphithan ^b,c^, Parichut Thummarati ^d,e^, Wanida Laiwattanapaisal ^d,e,*^

*^a^* Graduate Program in Clinical Biochemistry and Molecular Medicine, Department of Clinical Chemistry, Faculty of Allied Health Sciences, Chulalongkorn University, Bangkok, 10330, Thailand.

*^b^* Department of Biomedical Science, Faculty of Health and Society, Malmö University, 205 06, Malmö, Sweden

*^c^* Biofilms - Research Center for Biointerfaces, Malmö University, 205 06, Malmö, Sweden

*^d^* Centre of Excellence for Biosensors and Bioengineering (CEBB), Chulalongkorn University, Bangkok, 10330, Thailand

*^e^* Department of Clinical Chemistry, Faculty of Allied Health Sciences, Chulalongkorn University, Patumwan, Bangkok, 10330, Thailand

1. **The comparison of HT-29 cell morphology between 2D cell culture plate and 3D paper membrane**

To investigate the cell morphology of HT-29 cells on a 2D cell culture plate and 3D paper membrane using fluorescence imaging. In a 2D cell culture, cells exhibited a round and small shape and likely limited the *in vivo* conditions. In contrast, cells on a 3D paper membrane exhibited a more complex and likely to mimic the in vivo conditions, indicating a paper membrane provides the cell microenvironment that promotes cell growth in three dimensions as shown in supplementary Fig. S1A. In addition, the percentages of cell viability showed good viability with 98.80% and 99.21% of 2D and 3D cell culture, respectively, as shown in Supplementary Figure S1B.


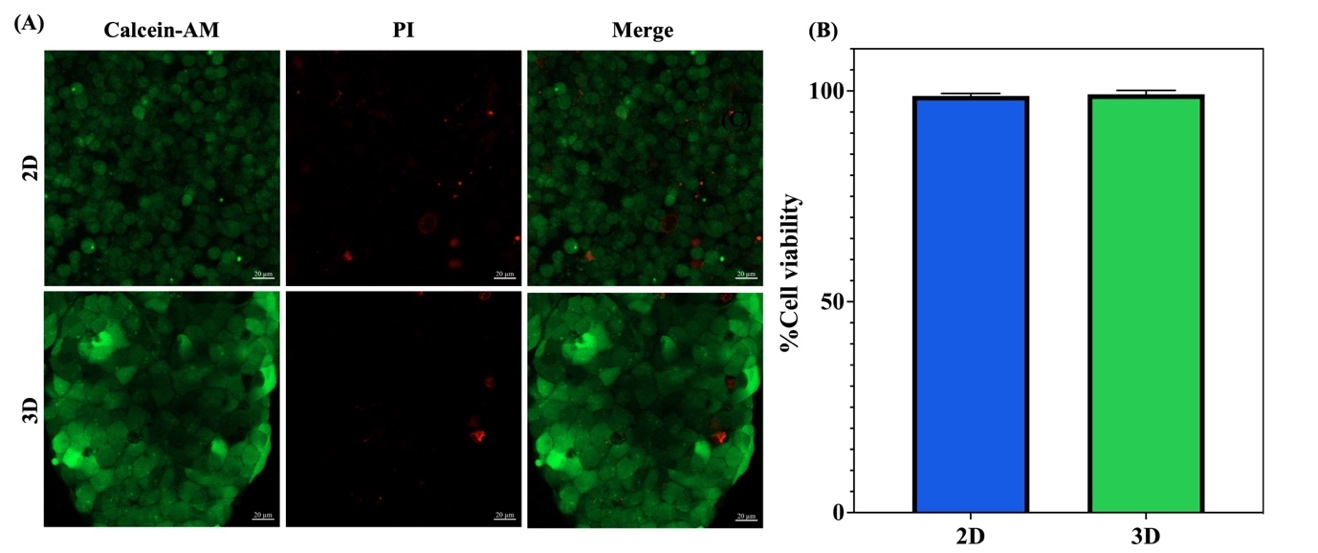


**Supplementary Figure S1** (A) The fluorescence images and (B) The percentage of cell viability of HT-29 cells on a 2D cell culture plate and 3D paper membrane. The scale bar is 20 µm. Data are presented as the mean ± standard deviation (SD) (n = 3).

1. **The permeability assay of a paper-based HT-29 cell membrane using a customizable 3D-printed transwell device.**

To investigate the permeability of HT-29 cells using FITC-dextran assay. HT-29 cells were cultured on a Matrigel-functionalized membrane for 48 h and then cells were treated with different conditions that contain a FITC-dextran dye. Aliquots of the culture medium from a basolateral compartment were collected at 1, 2, and 4 h, post-incubation, and then transferred into a 96-well back plate for fluorescence intensity measurement. The FITC-dextran assay exhibited an increase in the permeability of a Matrigel-functionalized membrane in all experimental conditions in time-dependent as shown in Supplementary Fig. S2.


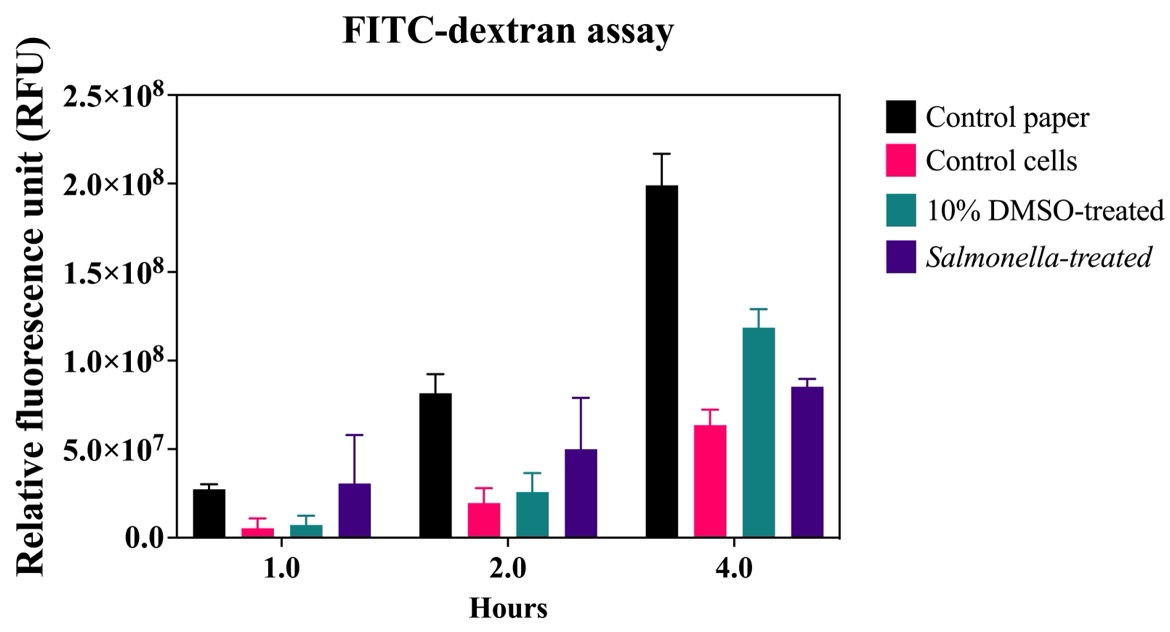


**Supplementary Figure S.2** The relative fluorescence unit (RFU) of a paper membrane at 1, 2, and 4 h, post-incubation of all conditions. Data are presented as the mean ± standard deviation (SD) (n = 3).
